# Workplace Involution and Employees’ Proactive Career Behavior: The Moderating Role of Construal Level

**DOI:** 10.3390/bs16030313

**Published:** 2026-02-25

**Authors:** Yali Jiang, Haiping Chen

**Affiliations:** 1 Faculty of Psychology, Beijing Normal University, Beijing 100875, China; 201321060046@mail.bnu.edu.cn; 2 Weifang Light and Salt Education Consulting Co., Ltd., Weifang 261000, China

**Keywords:** workplace involution, proactive career behavior, construal level

## Abstract

Workplace involution has become a widespread and salient phenomenon among employees in contemporary Chinese organizations. However, little is known about how workplace involution influences employees’ cognition and behaviors. Drawing on the Job Demands–Resources (JD-R) model and Construal Level Theory (CLT), this study investigated the effect of workplace involution on employees’ proactive career behavior and examined the moderating role of construal level. Study 1 employed a survey design with 284 full-time employees using validated measures of workplace involution, proactive career behavior, and construal levels. Study 2 adopted a scenario-based experimental design that manipulated workplace involution and construal level. Results from both studies consistently revealed that (1) workplace involution had a significant negative effect on employees’ proactive career behavior, and (2) construal level positively moderated this relationship. Specifically, a high construal level buffered the detrimental impact of workplace involution on proactive career behavior. These findings highlight the inhibitory mechanism of workplace involution on employees’ positive career behaviors and elucidate the cognitive boundary conditions underlying this effect. These results have theoretical and practical implications for promoting career proactivity in highly competitive organizational environments.

## 1. Introduction

In China, rapid economic development and social transformation have been accompanied by the widespread emergence of involution across social, educational, and occupational domains (Q. Chen & Zhang, 2022). Although the concept has been applied in diverse contexts and at different analytical levels, it is commonly used to describe intensified competition that generates limited or no proportional returns. In organizational research, involution is typically conceptualized as a social and organizational phenomenon characterized by “horizontal stability, repetitive effort, limited breakthrough, and untransformed dynamism” (Y. Zhang et al., 2024). Within this broader phenomenon, the workplace represents a particularly salient arena in which involution manifests (Q. Chen & Zhang, 2022). In the context of an oversupplied labor market, employment competition has intensified as the number of college graduates reached 12.22 million and youth unemployment rose to 18.9% (National Bureau of Statistics of China, 2025). At the same time, scarce high-quality occupational resources and the limited employment-absorbing capacity of emerging industries—despite technological advancement and the contraction of traditional sectors—have further exacerbated structural mismatches in the labor market (Herz & Van Rens, 2020). Under these conditions, employees often experience heightened levels of workplace involution, as they strive to avoid marginalization, demotion, or job loss (Dou et al., 2022). While the concept originated in the Chinese context, similar patterns of low-yield competition have been observed in other economies undergoing structural transformation, particularly since the onset of the COVID-19 pandemic (Kniffin et al., 2021), suggesting that workplace involution has broader relevance beyond China (Bennett & Mueller Loose, 2025).

Although workplace involution may co-occur with other well-established work-related constructs, such as role conflict or work overload, it is conceptually distinct from them. Role conflict refers to incompatible role expectations (Rizzo et al., 1970), whereas work overload emphasizes excessive job demands (Spector & Jex, 1998). Thus, while role conflict or work overload may accompany workplace involution, they do not fully capture its defining features. To deepen the understanding of workplace involution, researchers first delineated its conceptualization in organizational settings. One strand defines workplace involution from an “I feel” perspective. The research conceptualized it as employees’ subjective perception that intensified competition and increased effort in resource-constrained work environments do not yield commensurate returns (Q. Chen & Zhang, 2022; Cheng et al., 2024; Dou et al., 2022; Ma et al., 2025; Xia & Niu, 2025; Yin et al., 2023). Another strand approaches workplace involution from an “I do” perspective. These researchers defined it as a set of observable behavior patterns in which employees persistently expend large amounts of time and energy on low-efficiency or meaningless tasks, feel compelled to overexert themselves, and sacrifice their health merely to avoid falling behind or maintain superficial competitiveness (Dong et al., 2025; Yan et al., 2022). This study focuses on workplace involution from the “I feel” perspective, that is, employees’ workplace involution perception.

Existing research on workplace involution perception has primarily examined its consequences for employees’ attitudes, psychological states, and behaviors. Prior studies consistently show that workplace involution perception is associated with more negative or passive outcomes, including higher turnover intention and “lying-flat” tendencies (Q. Chen & Zhang, 2022; Ma et al., 2025; Xia & Niu, 2025; Yin et al., 2023), increased negative emotions such as anxiety, stress, and emotional exhaustion (Q. Chen & Zhang, 2022; Cheng et al., 2024; Dou et al., 2022; Ma et al., 2025), and reduced positive psychological states such as meaningfulness at work and well-being (Q. Chen & Zhang, 2022; Cheng et al., 2024). Taken together, the extant literature has largely focused on the adverse or withdrawal-related consequences of workplace involution.

However, this dominant focus leaves an important question unanswered: how does workplace involution perception influence employees’ proactive career behaviors? Drawing on the job demands–resources (JD-R) model, workplace involution can potentially undermine proactive career behaviors. Importantly, examining proactive career behaviors does not negate the adverse nature of workplace involution; rather, it highlights how employees may respond differently to the same hindrance demand. From a cognitive perspective, construal level is proposed as a key moderating factor that shapes how employees interpret workplace involution perception and, consequently, how it relates to proactive career behaviors. Accordingly, the present study integrates the JD-R model and construal level theory to examine both the direct effect of workplace involution perception on proactive career behaviors and the moderating role of construal level.

According to the JD-R model, job demands refer to aspects of work that require sustained physical and/or psychological effort and are therefore associated with physiological and psychological costs (Demerouti et al., 2001). Within this framework, job demands can be further differentiated into challenge and hindrance demands (Lesener et al., 2019). Whereas challenge demands may involve effortful activities that offer potential gains, hindrance demands represent work constraints that impede goal attainment and limit opportunities for growth and rewards. Prior research consistently shows that hindrance demands are particularly detrimental, as they are associated with negative affect, emotional exhaustion, and withdrawal-related outcomes (e.g., Mazzola & Disselhorst, 2019). According to the JD-R model, workplace involution perception can be conceptualized as a form of hindrance job demand. Its core features—such as perceived resource scarcity, blocked promotion opportunities, intensified internal competition, and compelled overwork—require sustained investments of time, cognitive effort, and emotional energy while offering limited opportunities for growth or meaningful returns (Junker et al., 2025; Mazzola & Disselhorst, 2019). Accordingly, workplace involution aligns more closely with hindrance than challenge demands within the JD-R framework (Peeters & Campos, 2023).

Within the JD-R framework, proactive career behavior represents a critical behavioral outcome that is highly sensitive to job demands and resource availability. According to the health impairment process of the JD-R model, high and sustained job demands reduce work engagement and proactive behavior (Bakker & Demerouti, 2017). In this study, proactive career behavior refers to self-initiated, anticipatory, and future-oriented actions through which individuals actively shape their career development and work environment (Parker & Collins, 2010). JD-R-based research shows that proactive career behavior typically flourishes in contexts with ample resources and higher engagement, whereas it is often inhibited when hindrance demands are high and resources are low (Nguyen et al., 2021). Resource scarcity and promotional bottlenecks in workplace involution erode employees’ perceived controllability in achieving career goals. Meaninglessness and inefficient busyness in workplace involution undermine the sense of work meaningfulness. Prolonged, compelled competition and health-sacrificing overwork of workplace involution exacerbate emotional exhaustion and cynicism (e.g., Q. Chen & Zhang, 2022; Yin et al., 2023). Evidence also indicates that individuals with a lower subjective socioeconomic status tend to experience low self-control and self-efficacy, which can curtail personal resources from engaging in proactive behaviors (Dong et al., 2025). Taken together, these arguments suggest that proactive career behavior is particularly vulnerable to hindrance job demands such as workplace involution, making it a theoretically meaningful outcome within the JD-R framework. In conclusion, based on the JD-R model, workplace involution may exert a crowding-out effect on proactive career behaviors.

According to the JD-R model, the negative effects of hindrance job demands on positive work behaviors are not inevitable but are moderated by personal resources. Bakker and Demerouti (2024) explicitly include cognitive and self-regulatory capacities as personal resources, arguing that such resources help sustain engagement and shape how individuals appraise job demands. Accordingly, construal level, as a cognitive personal resource, may moderate the relationship between workplace involution perception and proactive career behavior.

Construal Level Theory (CLT) suggests that individuals can represent events at various levels of abstraction (Trope & Liberman, 2003). High-level construal emphasizes abstract, goal-relevant “why” aspects, whereas low-level construal focuses on concrete, contextual “how” aspects (Bar-Anan et al., 2006). Empirical evidence indicates that high construal levels enhance self-control and persistence in difficult but meaningful tasks (Liberman & Trope, 2020). From a JD-R perspective, this future-oriented and goal-focused processing style can function as a personal resource, potentially buffering the adverse effects of hindrance demands (Bakker & Demerouti, 2024).

Although the present study does not directly test specific psychological mechanisms, prior research offers three complementary rationales for why construal level may buffer the effects of workplace involution. From a meaning-making perspective, high construal levels help employees interpret workplace involution in terms of higher-order goals and long-term development, thereby sustaining commitment to proactive career efforts (Liberman & Trope, 2020). From an affective self-regulation perspective, high construal levels increase psychological distance from stressors, reducing negative affect and strain and thus preserving resources for proactive behavior (Abraham et al., 2023; Bakker & Demerouti, 2024). From a coping perspective, high construal levels foster problem-focused coping, enabling more constructive responses in high-demand, low-resource contexts (Maglio & Trope, 2019; Van Zyl et al., 2025). Taken together, construal level may function as a key personal resource that shapes employees’ responses to workplace involution, thereby moderating its relationship with proactive career behavior.

**Figure 1:**
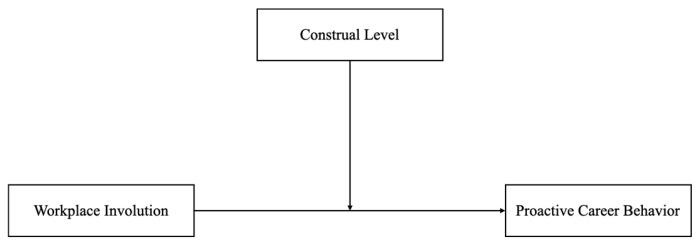
The proposed model.

Conceptually, workplace involution perception represents a hindrance job demand, proactive career behavior serves as the focal behavioral outcome, and construal level operates as a personal resource that shapes employees’ motivational, affective, and coping responses to workplace involution. Under high levels of involution, employees with a high construal level are more likely to adopt a more abstract and future-oriented perspective, which buffers the negative impact of workplace involution on proactive career behavior. In contrast, employees with a low construal level tend to focus on immediate constraints and affective reactions, thereby intensifying the detrimental effect of workplace involution on proactive career behavior. Accordingly, construal level is expected to moderate the negative relationship between workplace involution perception and proactive career behavior. As shown in Figure 1, the proposed model illustrates the relationship between workplace involvement and proactive career behavior.

To address the growing prevalence of workplace involution and its implications for employees’ career-related behaviors, the present study pursues two primary research objectives. First, it aims to examine whether employees’ workplace involution are associated with proactive career behavior. Second, it seeks to investigate whether construal level serves as a key personal resource that moderate the potential negative effects of workplace involution.

Accordingly, this study is guided by the following research questions:

**RQ1:**  *Is workplace involution perception negatively associated with employees’ proactive career behavior?*

**RQ2:**  *Does construal level moderate the relationship between workplace involution perception and proactive career behavior, such that the negative effect is weaker at higher levels of construal?*

To answer these research questions, we adopted a multi-study design. Study 1 employed a cross-sectional survey to examine baseline associations among workplace involution perception, construal level, and proactive career behavior. Study 2 used a scenario-based experimental design that manipulated workplace involution and construal level to establish causal inferences. Based on the above theoretical framework and empirical rationale, we propose the following hypotheses.

**H1:**  *Workplace involution negatively correlates with proactive career behavior.*

**H2:**  *Construal level moderates the relationship workplace involution perception and proactive career behavior, such that the negative relationship is weaker when employees’ construal level is high rather than low.*

## 2. Study 1

### 2.1. Participants

A total of 284 participants were recruited using the Credamo platform. Each participant received a monetary compensation of 5 RMB upon careful completion of the survey. Responses were screened based on completion time and attention check questions, resulting in 284 valid responses. According to the Measures for Ethical Review of Research Involving Humans, jointly issued by the Ministry of Education and other relevant departments of China (effective in 2023), research involving non-identifiable public data or fully anonymized human data is exempt from formal ethics committee review. This regulation explicitly states that studies using anonymized data may be exempt from ethical review procedures. As this study was conducted through a completely anonymous online survey, no formal ethics approval was required. The demographic characteristics of the participants are summarized in Table 1.

### 2.2. Design and Procedure

Study 1 employed a cross-sectional survey design. Data were collected via an online survey platform in December 2025.

### 2.3. Measures

Workplace involution was measured using the Workplace Involution Scale developed by Q. Chen and Zhang (2022). The original scale consists of four dimensions—sense of resource scarcity, sense of compelled commitment, sense of futility of effort, and sense of negative experience—with four items per dimension (16 items in total). In the present study, workplace involution was conceptualized as an overall perceptual construct rather than as a set of distinct dimensions. Accordingly, and following prior measurement practices that adopt factor-loading–based item selection to achieve a parsimonious representation of multidimensional constructs (e.g., C. Chen et al., 2025), we selected two items with the highest standardized factor loadings and strongest conceptual representativeness from each dimension. The factor loadings used for item selection were derived from the original scale development and validation study by Q. Chen and Zhang (2022), ensuring that all four dimensions were retained in the reduced scale.

We acknowledge that the original 16-item scale is not excessively long and remains suitable for many research contexts. Our decision to adopt an eight-item version was therefore not driven by scale length alone, but by the analytical focus on workplace involution perception and the need for measurement parsimony and consistency across a multi-study design. Although no separate pre-test was conducted specifically for item reduction, the psychometric properties of the reduced scale were re-examined using the current study sample. As reported in the Results Section 2.4, exploratory and confirmatory factor analyses indicated satisfactory construct validity, and the scale demonstrated excellent internal consistency (Cronbach’s alpha = 0.93). An example item is, “I feel that the current industry in which I work is economically depressed.” All items were rated on a five-point Likert scale ranging from 1 (“strongly disagree”) to 5 (“strongly agree”), with higher scores indicating a higher perceived level of workplace involution.

Proactive career behaviors were assessed using the Career Engagement Scale developed by Hirschi et al. (2014). An example item is: “Voluntarily participated in further education, training, or other events to support your career.” Participants rated each item on a five-point Likert scale (1 = “strongly disagree,” 5 = “strongly agree”), with higher scores indicating a higher level of proactive career behavior. In the present study, the scale demonstrated good internal consistency with a Cronbach’s alpha of 0.80.

Construal level was measured using the brief and context-general Construal Level Scale developed by Venus et al. (2019). The scale includes three items, such as “I am focused on the big picture rather than on details.” Responses were recorded on a five-point Likert scale ranging from 1 (“strongly disagree”) to 5 (“strongly agree”), with higher scores reflecting a higher construal level. In the present study, the scale demonstrated good internal consistency, with a Cronbach’s alpha of =0.80.

### 2.4. Results

Exploratory factor analysis (EFA) and confirmatory factor analysis (CFA) were employed to validate the instruments adopted in the present study. EFA with varimax rotation was conducted to investigate the construct validity of each subscale. The KMO value was 0.87, and Bartlett’s test of sphericity was 2806.62 (*p* < 0.001). Furthermore, the structure explained 56.57% of the total variance in the model. Table 1 lists the factor loadings of the items. Collectively, these results suggest that this solution is a reasonable interpretation of the present survey (Hair et al., 2010).

A CFA was conducted to validate the instruments. The measurement model was assessed for goodness-of-fit, construct reliability, and construct validity. Goodness-of-fit was determined based on GFI, CFI, TLI, and RMSEA. All model fit statistics were within the acceptable ranges (*χ*^2^/*df* = 2.07 < 3, *GFI* = 0.91 > 0.90, *CFI* = 0.94 > 0.90, *TLI* = 0.93 > 0.90, *RMSEA* = 0.06 < 0.08, *SRMR* = 0.09 < 0.1) (Hair et al., 2010), which demonstrates that the measurement model exhibits satisfactory values.

The construct reliability of the model was determined using convergent validity (AVE) and composite reliability (CR). As shown in Table 2, both workplace involution and construal level scales’ average variance extracted (AVE) were greater than 0.5, which was satisfactory (Segars, 1997). The composite reliability values of the workplace involution and construal level scales were all over 0.7, demonstrating satisfactory reliability (Nunnally & Bernstein, 1994). However, the AVE of the proactive career behavior scale’s AVE was 0.32. This scale was originally developed as a unidimensional construct. Although the AVE (0.32) was slightly below 0.50, the composite reliability (0.81) indicated strong internal consistency. According to Fornell and Larcker (1981), when AVE is less than 0.50 but CR exceeds 0.60, convergent validity remains acceptable. Therefore, the measurement model of this single-factor scale was considered acceptable.

As presented in Table 3, the square roots of AVE were compared with the correlations among the latent variables to evaluate discriminant validity (Fornell & Larcker, 1981). All latent correlations were less than the corresponding AVE square root, which was satisfactory. Overall, these results indicated that the proposed research model had a good fit.

#### 2.4.1. Descriptive Statistics and Correlation Analysis

Table 4 shows a significant negative correlation between workplace involution and proactive career behavior (*r* = −0.21, *p* < 0.01), a significant positive correlation between workplace involution and construal level (*r* = 0.13, *p* < 0.05), and a significant negative correlation between workplace involution and monthly income. There is a significant positive correlation between construal level and proactive career behavior (*r* = 0.21, *p* < 0.01).

Regarding the control variables, proactive career behavior shows modest associations with gender, educational attainment, and monthly income, indicating that these demographic factors are related to career proactivity and therefore warrant statistical control in subsequent analyses. Importantly, the observed correlations do not alter the hypothesized relationships among the focal variables. These control variables were included to rule out alternative explanations rather than to test substantive hypotheses.

#### 2.4.2. Hypothesis Tests

A moderation analysis was conducted using Model 1 in PROCESS v3.4, with all variables standardized except for the dependent variable. After controlling for gender, education, and monthly income, the results are presented in Table 5.

According to the results of Model 1, workplace involution was significantly negatively associated with proactive career behavior (*β* = −0.20, *p* < 0.01), supporting Hypothesis 1.

As shown in Model 3, the interaction term between workplace involution and construal level significantly predicted proactive career behavior (*β* = 0.20, *p* < 0.01).

**Figure 2:**
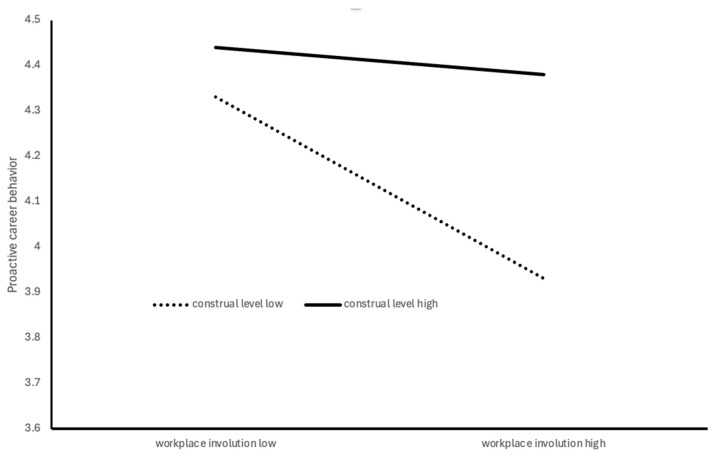
The moderating role of construal level.

The simple slope analysis revealed that workplace involution significantly negative correlated with proactive career behavior at low (−1 SD; *β* = −0.190, *p* < 0.001) and mean levels of construal level (*β* = −0.109, *p* < 0.001), but the effect was nonsignificant at high levels of construal level (+1 SD; *β* = −0.029, *p* = 0.380). These results suggest that higher construal levels attenuate the negative effect of workplace involution on proactive career behavior. Figure 2 graphically depicts the interaction pattern, showing how the relationship between workplace involution and proactive career behavior varies across levels of construal level.

### 2.5. Discussion

The results of Study 1 indicated that workplace involution was significantly positively associated with employees’ proactive career behavior and that construal levels significantly moderated this relationship. Specifically, compared with employees with a low construal level, the negative association between workplace involution and proactive career behavior was weaker among those with a high construal level. This finding aligns with previous research suggesting that workplace involution often leads to negative employee outcomes (e.g., Q. Chen & Zhang, 2022; Dou et al., 2022; Ma et al., 2025) and is also consistent with the JD-R model (e.g., Bakker & Demerouti, 2024). On the one hand, the results suggest that workplace involution is primarily perceived by employees as a job demand that requires internal resource expenditure and functions as a form of job hindrance (Yin et al., 2023; W. Zhang et al., 2025). On the other hand, this study is the first to demonstrate that construal level serves as an important supportive personal resource that shapes how employees evaluate involution. A higher construal level can buffer the detrimental impact of workplace involution on proactive career behavior (Bakker & Demerouti, 2024). Although Study 1 adopted a cross-sectional design, it provided preliminary evidence of the relationships among these variables. To further clarify the causal relationships, Study 2 employed a scenario-based behavioral experiment by manipulating perceived levels of workplace involution and construal, and measuring proactive career behavior.

## 3. Study 2

### 3.1. Participants

Before data collection, an a priori power analysis was conducted using GPower 3.1.9.7 (Faul et al., 2007) to determine the minimum sample size required to detect a medium effect. The parameters were set according to conventional standards in behavioral research: significance level α = 0.05, statistical power (1 − β) = 0.80, and an expected medium effect size of f = 0.25 (Cohen, 1988). Under these specifications and assuming equal allocation across conditions, GPower estimated that a total sample size of *N* = 128 (approximately 32 participants per cell) would be sufficient to detect a medium effect with adequate power.

Data were collected through the Credamo platform, and eligibility was restricted to currently employed participants. According to the Measures for Ethical Review of Research Involving Humans, jointly issued by the Ministry of Education and other relevant departments of China (effective in 2023), research involving non-identifiable public data or fully anonymized human data is exempt from formal ethics committee review. This regulation explicitly states that studies using anonymized data may be exempt from ethical review procedures. As this study was conducted through a completely anonymous online survey, no formal ethics approval was required. After excluding invalid responses based on completion time and one attention-check item, 209 valid cases were retained for analysis. At least 49 participants were included for each experimental condition. The participants’ demographic information is presented in Table 6.

### 3.2. Design and Procedure

#### 3.2.1. Experimental Design

This study employed a 2 × 2 between-subjects experimental design, with workplace involution (high vs. low) and construal level (high vs. low) as the independent variables, and proactive career behavior as the dependent variable.

#### 3.2.2. Manipulations

The manipulation of perceived workplace involution was adapted from the scenario task developed by Y. Zhang et al. (2024). However, the original scenario of Y. Zhang et al. (2024) focused solely on overtime. To better capture the multidimensional nature of workplace involution, this study reconstructed a scenario task based on the four dimensions proposed by Q. Chen and Zhang (2022): sense of resource scarcity, sense of compelled commitment, sense of futility of effort, and sense of negative experience. Two situational vignettes were designed to manipulate high and low levels of perceived workplace involution.

Manipulation of the construal level was conducted according to Trope and Liberman’s (2003) procedure. In the high-construal-level condition, participants were instructed to reflect on why they performed a given behavior, encouraging them to focus on its broader purpose, meaning, and underlying reasons. In the low construal-level condition, participants were asked to think about how they performed the behavior, directing their attention to the concrete steps, processes, and details involved (see Appendix A).

### 3.3. Measurement

The measurement of proactive career behavior was identical to that in Study 1. However, to enhance participants’ immersion in the experimental scenario, each item was preceded by an instruction phrase: “Based on the scenario you have just read, please indicate the extent to which you would engage in the following behaviors.”

The manipulation check for perceived workplace involution was conducted using six items selected from the Workplace Involution Scale developed by Q. Chen and Zhang (2022), which was consistent with Study 1.

The manipulation check for construal level was identical to that in Study 1.

### 3.4. Results

#### 3.4.1. Manipulation Checks

Perceived workplace involution scores were used to examine the effectiveness of the involution manipulation. The results indicated that participants in the high-involution condition (*M* = 3.80, *SD* = 0.73) reported significantly higher levels of perceived workplace involution than those in the low-involution condition (*M* = 1.74, *SD* = 0.44; *t* = 24.75, *p* < 0.001, Cohen’s *d* = 0.61). These findings suggest that the workplace involution manipulation was successful.

Similarly, construal-level scores were used to test the effectiveness of construal-level manipulation. Participants in the high-level construal condition (*M* = 3.67, *SD* = 0.47) reported significantly higher construal-level scores than those in the low-level construal condition (*M* = 3.52, *SD* = 0.39), *t* = 2.06, *p* < 0.05, Cohen’s *d* = 0.45. Thus, the construal level manipulation was effective.

#### 3.4.2. Normal Distribution Test

Before conducting a two-way between-subject analysis of variance (ANOVA), the normality assumption was examined. Given that each group contained more than 44 participants, Kolmogorov–Smirnov tests were conducted on the unstandardized residuals, supplemented by inspection of skewness and kurtosis values. Although formal normality tests indicated some deviations from exact normality, the absolute values of skewness (2.29) and kurtosis (7.37) were within commonly accepted thresholds (|skewness| < 3, |kurtosis| < 10), suggesting that the residuals were approximately normally distributed (Kline, 2011). At the same time, previous studies generally believe that ANOVA is robust to violations of normality assumptions (Field, 2018). Therefore, a Univariate Analysis of Variance (UNIANOVA) was considered appropriate for this study. Resampling-based bootstrap procedures were used to assess the robustness of the results as recommended by Pek et al. (2017). Specifically, bias-corrected and accelerated (BCa) bootstrap confidence intervals were computed based on 5000 re-samples.

#### 3.4.3. Proactive Career Behavior

Levene’s tests were conducted to examine the homogeneity of variances. The results indicated that the assumption of equal variances was violated, *F*(3, 205) = 21.70, *p* < 0.001. To address this violation, bootstrapping procedures were employed to assess the robustness of the results (Pek et al., 2017).

A 2 (workplace involution: high vs. low) × 2 (construal level: high vs. low) UNIANOVA was performed on the proactive career behavior. The results are presented in Table 1. The results revealed a significant main effect of workplace involution perception, *F*(1, 205) = 6.18, *p* = 0.02, partial *η*^2^ = 0.029. Employees who perceived a high level of workplace involution exhibited significantly lower levels of proactive career behavior than those who perceived a low level of workplace involution. The main effect of construal level was not significant, *F*(1, 205) = 0.62, *p* = 0.43, partial *η*^2^ = 0.001.

**Figure 3:**
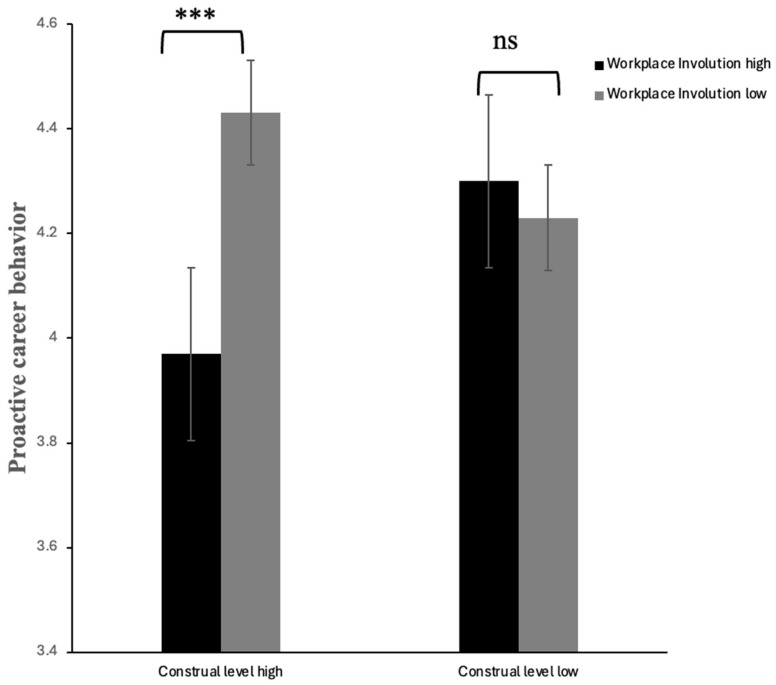
The effect of construal level on employees’ proactive career behavior in high and low workplace involution. Note. *** *p* < 0.001; ns = not significant.

Importantly, the interaction between involution and explanation framing was significant, *F*(1, 205) = 10.58, *p* = 0.001, partial *η*^2^ = 0.05. Given the violation of the homogeneity of variance assumption, simple effects were examined using Welch’s *t*-tests. Among employees with a high construal level, proactive career behavior was significantly lower under high workplace involution than under low workplace involution, t(72.15) = −3.74, *p* < 0.001, Cohen’s *d* = −0.65. No significant difference was observed between proactive career behaviors in high and low workplace involution perception among employees with low construal levels, t(88.18) = 0.90, *p* < 0.001, Cohen’s *d* = 0.619. Bootstrap analyses based on 5000 re-samples yielded confidence intervals consistent with the parametric results, indicating that the findings were robust against violations of the homogeneity of variance assumption. The results are shown in Table 7 and Figure 3.

### 3.5. Discussion

Study 2 extended the findings of Study 1 by employing experimental vignette methodology (EVM; Aguinis & Bradley, 2014) to establish causal relationships among workplace involution, construal level, and employees’ proactive career behavior. By manipulating both the independent variable (workplace involution) and the moderator (construal level), the study provided stronger and convergent evidence for the proposed framework. Manipulation checks confirmed that both workplace involution and construal level were successfully manipulated, thereby offering diverse and valid methodological options for future research (W. Zhang et al., 2025).

The results of Study 2 indicated a significant main effect of workplace involution: employees experiencing high workplace involution exhibited significantly lower proactive career behavior than those experiencing low workplace involution. More importantly, the interaction between workplace involution and construal level was significant. Simple effects analyses revealed that, within the high-construal-level condition, employees with low workplace involution exhibited significantly higher proactive career behavior than those with high workplace involution. In contrast, within the low construal-level condition, no significant differences in proactive career behavior were observed between employees with high and low workplace involution.

These findings are consistent with theoretical expectations that high workplace involution elicits a short-term, reactive mindset that suppresses future-oriented, proactive behaviors (Bakker & Demerouti, 2024). Moreover, the experimental design strengthens causal inference by reducing potential confounds inherent in the cross-sectional survey design used in Study 1. Thus, the findings not only replicate but also extend the correlational evidence from Study 1, offering stronger support for the moderating role of construal level. Taken together, the two studies provide complementary evidence that workplace involution and employees’ cognitive framing jointly shape proactive career engagement.

## 4. General Discussion

Study 1 (a cross-sectional survey) and Study 2 (a scenario-based experiment) were jointly conducted to examine the relationships among workplace involution, construal level, and employees’ proactive career behavior. The main findings indicate that workplace involution exerts a negative impact on employees’ proactive career behavior, while construal level serves as a crucial buffering variable that positively moderates this negative relationship. It is important to note that the present findings are based on a specific organizational and cultural context, in which workplace involution is particularly salient, and thus do not imply that involution affects all employees uniformly.

First, workplace involution was found to be significantly and negatively associated with proactive career behavior, suggesting that employees who perceive higher levels of workplace involution are less likely to engage in proactive career activities. This finding aligns with prior research documenting the detrimental effects of involution in organizational contexts. For example, perceived involution has been shown to positively predict turnover intention (Q. Chen & Zhang, 2022). Involution may also lead to emotional exhaustion and work-related anxiety, which in turn trigger “Tang Ping” (lying flat) behaviors (e.g., Yin et al., 2023). According to the JD-R model, high workplace involution is associated with reduced perceptions of development opportunities, lower organizational support, and diminished fairness (e.g., Xia & Niu, 2025). These characteristics not only deplete employees’ self-regulatory resources through the health impairment process but also undermine their sense of meaning and intrinsic motivation through the motivational process, thereby accelerating a spiral of resource loss (Kim & Beehr, 2022).

Workplace involution, as a hindrance demand, is especially taxing because it consumes cognitive resources by creating a cognitive conflict. Specifically, employees are caught between the need to invest increasing effort to remain competitive and the perception that such effort yields diminishing or uncertain returns. It can trigger feelings of frustration and helplessness (Mazzola & Disselhorst, 2019). These experiences place strain on individuals’ cognitive resources, fostering rumination, which is characterized by narrowed attentional focus and difficulty disengaging from immediate work-related problems, thereby undermining long-term goal-directed thinking (Janurek et al., 2024). Consequently, individuals experiencing greater workplace involution are less likely to engage in proactive career behavior, which requires the ability to focus on long-term goals and sustain motivation over time. In this sense, involution can narrow employees’ cognition, making it harder for them to view their work environment as a space for growth and opportunity. Given that proactive career behavior requires individuals to invest additional resources and maintain a high level of autonomous motivation (Lent et al., 2022), employees experiencing stronger workplace involution are less likely to engage in such behaviors.

Second, construal level moderates the relationship between workplace involution and employees’ proactive career behavior. Specifically, a higher construal level buffers the negative impact of workplace involution on proactive career behavior. From a JD-R perspective, personal resources are capacities that help individuals manage job demands, protect cognitive and emotional energy, and sustain goal-directed behavior under stress. In this regard, construal level operates as a protective resource by counteracting the cognitive depletion induced by workplace involution.

High levels of construal promote abstract, higher-order interpretation of work experiences, enabling employees to situate involution within broader career trajectories and long-term developmental goals (Trope & Liberman, 2010). By reconstructing the meaning of involution-related demands in relation to personal values and future aspirations (Q. Zhang et al., 2022), a high level of construal reduces short-term fixation and preserves a sense of purpose, thereby mitigating cognitive myopia and sustaining commitment to proactive career behavior. High levels of construal increase psychological distance from stressors, reducing emotional intensity and strain during stress appraisal (Abraham et al., 2023). Consistent with the JD-R health-impairment process, this reduction in negative affect slows the depletion of cognitive and emotional resources (Bakker & Demerouti, 2024), mitigating stress-related fatigue and preserving resources for proactive behavior. A high level of construal fosters problem-focused coping strategies (Maglio & Trope, 2019), enabling employees to address involution not as an immediate threat but as a structural condition that can be managed through skill development, career planning, or job crafting. Prior research supports this view, showing that higher construal levels weaken the link between work stress and maladaptive behaviors such as cyberloafing, while promoting constructive forms of self-initiated change (Demerouti & Bakker, 2023; Van Zyl et al., 2025). Moreover, consistent with evidence that construal level shapes responses to situational work stress, it can buffer detrimental effects on work engagement and attenuate stress-related insecurity (Wang et al., 2018). In addition, high-level construal reduces perceived threat and negative emotional intensity, thereby slowing resource depletion and enabling employees to retain resources for other goal-directed activities (Hua & Mi, 2025). Taken together, high construal level serves as a cognitive personal resource that bridges abstraction (CLT) and operational resilience (JD-R) by broadening attentional scope, regulating affective responses, and enabling problem-focused coping. Through these mechanisms, high construal levels protect employees from stress-induced cognitive myopia and preserve the cognitive and motivational resources necessary for proactive career behavior under workplace involution.

An additional contribution of this study lies in its multi-study design. By combining a cross-sectional survey (Study 1) with a scenario-based experiment (Study 2), the findings benefit from both ecological validity and stronger causal inference. This design enhances the robustness and credibility of the conclusions by demonstrating consistent patterns across complementary methodological approaches.

### 4.1. Theoretical Implications

The findings from this study provide several key theoretical contributions to the understanding of workplace involution, construal level, and proactive career behavior. First, we extend the JD-R model by demonstrating that workplace involution acts as a significant hindrance demand, depleting employees’ proactive career behaviors. Second, this study advances CLT by illustrating that construal level serves as a crucial personal resource that buffers the negative effects of workplace involution. High construal levels promote abstract thinking, allowing employees to reframe workplace involution within broader career trajectories and long-term developmental goals. Third, this study contributes to and extends the proactive career behavior literature by identifying workplace involution as a critical contextual antecedent. Prior research on proactive career behavior has largely emphasized individual traits, career competencies, and supportive organizational factors, while paying relatively less attention to how adverse and resource-depleting work environments may inhibit such behavior (Jiang et al., 2023). By conceptualizing workplace involution as a hindrance demand, our findings enrich the proactive career behavior literature by highlighting how structural and competitive work contexts can undermine employees’ willingness and capacity to proactively manage their careers.

### 4.2. Practical Implications for Organizational Human Resource Management

Implications for corporate managers. The findings are particularly relevant for highly competitive organizational environments characterized by intense internal competition and zero-sum performance pressures. In such contexts, workplace involution is more likely to function as a hindrance demand that depletes employees’ cognitive resources and discourages proactive career behavior. Accordingly, organizations should prioritize curbing zero-sum internal competition and redesigning work systems that lock employees into high input–low return–low control cycles (Fernandez de Henestrosa et al., 2023). However, beyond structural interventions, managers can actively cultivate employees’ high-level construal as a cognitive protective resource. For example, career development interviews, coaching conversations, and performance feedback can be deliberately framed around long-term career trajectories, transferable skills, and personal values rather than short-term performance metrics alone. Such practices help employees cognitively distance themselves from immediate competitive pressures and reconnect daily tasks with broader career goals, thereby counteracting stress-induced cognitive myopia (Bakker & Demerouti, 2024). In addition, organizations may implement structured reflection interventions—such as goal-mapping workshops, career crafting programs, or job redesign initiatives—that encourage employees to reinterpret involution-related demands as part of a longer-term developmental process. By systematically embedding abstraction-oriented cues into HR practices, managers can strengthen employees’ construal levels and prevent behavioral paralysis in highly involuted environments.

Implications for employees. On the one hand, employees can consciously cultivate a high-level construal mindset by reframing involution as a temporary phase within their career trajectory. By clarifying long-term career goals and engaging in proactive behaviors, they may transform current pressure into future competitive advantages (Martínez-Díaz et al., 2020). On the other hand, employees should actively seek social support and developmental resources to build relatively independent growth pathways, even within highly involuted environments.

### 4.3. Limitations and Future Research Directions

This study has several limitations that warrant further investigation. First, although combining a cross-sectional survey with a scenario-based experiment provides complementary insights across naturalistic and controlled contexts, the cross-sectional design limits strong causal inference (Ployhart & Vandenberg, 2010), and the scenario-based manipulation in Study 2 may not fully capture the chronic stress and sustained exhaustion associated with workplace involution. Accordingly, the experimental findings should be interpreted as reflecting behavioral intentions rather than actual behavior under prolonged involution. Future research could strengthen both causal inference and ecological validity by adopting longitudinal or multi-wave designs, incorporating multi-source data, and employing field or experience sampling methods (Bakker & Demerouti, 2024).

Second, reliance on self-reported data may introduce common method bias. The data were collected via an online platform, which could further exacerbate this bias and limit the generalizability of the findings. In addition, the relatively young and likely urban-centric sample reflects early-career employees who may experience workplace involution differently from more established workers. Accordingly, the findings should be interpreted with caution, and future research is encouraged to replicate the model using more diverse samples across career stages and organizational contexts.

Third, in Study 2, proactive career behavior exhibited greater variance in the high workplace involution condition, suggesting heterogeneous responses to the manipulation. This variability may reflect individual differences in sensitivity to competitive or resource-constrained work environments. Although variance-robust analyses were employed, future research could improve manipulation precision through more tightly controlled scenarios or targeted manipulation checks.

Fourth, this study did not examine the mediating mechanisms through which workplace involution affects proactive career behavior. Drawing on the JD-R model, future studies could include mediators such as basic psychological need frustration, perceived work meaningfulness, and perceived career control to better elucidate the underlying processes.

## 5. Conclusions

Drawing on the Job Demands–Resources model and Construal Level Theory, this research examined how workplace involution influences employees’ proactive career behavior using a cross-sectional survey and an experimental vignette methodology. Across both studies, workplace involution emerged as a hindrance job demand that was negatively associated with proactive career behavior. Importantly, a higher construal level buffered these detrimental effects: employees with a low level of construal were less likely to reduce proactive career behavior in response to workplace involution. The present findings underscore the importance of examining a broader set of personal and organizational resources that enable career proactivity under adverse work conditions.

## Figures and Tables

**Table 1:** Sample demographics (study 1).

	Options	Frequency	Percentage (%)
Gender	Male	102	35.92
	Female	182	64.08
Career type	Self-employed/Other	88	30.99
	Public institution/State-owned enterprise	149	52.46
	Private enterprise	27	9.51
	Foreign-funded enterprise	20	7.04
Education	Associate degree or below	42	14.79
	Bachelor’s degree	174	61.27
	Master’s degree or above	68	23.94
Monthly income	Below 5000 RMB	42	14.79
	5001–10,000 RMB	140	49.30
	10,001–20,000 RMB	82	28.87
	Over 20,000 RMB	20	7.04
Age	Below 30	143	50.35
	31–40	114	40.14
	40 above	27	9.51
	Total	284	

**Table 2:** Exploratory factor analysis results (only factor loadings greater than 0.5 are shown).

	Component		
	1	2	3
Workplace involution 1	0.79		
Workplace involution 2	0.87		
Workplace involution 3	0.76		
Workplace involution 4	0.80		
Workplace involution 5	0.81		
Workplace involution 6	0.79		
Workplace involution 7	0.88		
Workplace involution 8	0.87		
Construal level1		0.87	
Construal level2		0.75	
Construal level3		0.86	
PCB1			0.74
PCB2			0.67
PCB3			0.60
PCB4			0.73
PCB5			0.62
PCB6			0.53
PCB7			0.55
PCB8			0.60
PCB9			0.63

Note: PCB = Proactive Career Behavior.

**Table 3:** Results of construct reliability and validity.

	Convergent Validity AVE	Composite Reliability CR
Workplace involution	0.58	0.81
Proactive career behavior	0.32	0.81
Construal level	0.64	0.94

**Table 4:** Descriptive statistics and correlations between variables.

Variables	M	SD	1	2	3
1. Workplace involution	3.29	1.10	-		
2. Proactive career behavior	4.28	0.45	−0.21 **	-	
3. Construal level	3.89	0.91	0.13 *	0.21 **	-
4. Gender	1.64	0.48	0.02	−0.12 *	−0.05
5. Career type	1.92	0.83	0.05	−0.07	−0.06
6. Education	2.09	0.80	−0.05	0.18 *	0.12 *
7. Monthly income	2.28	0.80	−0.16 **	0.15 *	0.16 *
8. Age	2.64	0.80	−0.09	0.06	0.07

Note: * *p* < 0.05; ** *p* < 0.01.

**Table 5:** Analysis Results of the Moderating Role of Construal Level.

	Proactive Career Behavior (Model 1)		Proactive Career Behavior (Model 2)		Proactive Career Behavior (Model 3)	
	*β*	*t*	*β*	*t*	*β*	*t*
Gender	−0.11	−1.92	−0.10	−1.81	−0.10	−1.68
Education	0.15	2.44 *	0.13 *	2.24 *	0.11	1.85
Monthly income	0.06	0.93	0.02	0.36	0.02	0.38
Workplace involution	−0.20 **	−3.36 **	−0.23 **	−4.03	−0.25 **	−4.36
Construal level			0.22 **	3.80	0.20 **	3.45
Workplace involution × Construal level						
*R* ^2^	0.09		0.13		0.17	
*F*	*F*(4, 279) = 6.85 **		*F*(5, 278) = 14.43 **		*F*(6, 277) = 9.45 **	

Note: * *p* < 0.05; ** *p* < 0.01.

**Table 6:** Sample demographics (study 2).

	Options	Frequency	Percentage (%)
Gender	men	98	46.89
	women	111	53.11
Career type	Self-employed/Other	13	6.22
	Public institution/State-owned enterprise	49	23.44
	Private enterprise	126	60.29
	Foreign-funded enterprise	21	10.05
Education	Associate degree or below	21	10.05
	Bachelor’s degree	152	72.73
	Master’s degree or above	36	17.22
Experimental conditions	High involution perception × High construal level	63	30.14
	High involution perception × Low construal level	44	21.05
	Low involution perception × High construal level	54	25.84
	Low involution perception × Low construal level	48	22.97
Total		209	

**Table 7:** Proactive career behavior of employees with high and low construal levels in high and low workplace involution situations.

	Construal Level High (n = 117)		Construal Level Low (n = 92)	
	*M*	*SD*	*M*	*SD*
Workplace Involution high (n = 107)	3.97	0.98	4.30	0.34
Workplace Involution low (n = 102)	4.43	0.25	4.23	0.60

## Data Availability

The data will be available upon request.

## Abbreviations

The following abbreviations are used in this manuscript:

JD-R	Job Demand–Resources Model
CLT	Construal Level Theory
EFA	Exploratory Factor Analysis
CFA	Confirmatory Factor Analysis
AVE	Average Variance Extracted
ANOVA	Analysis of Variance
UNIANOVA	Univariate Analysis of Variance
EVM	Experimental Vignette Methodology

## Appendix A

### Appendix A.1. High Involution Condition

Please imagine that you have just joined a new company with generally attractive benefits and working conditions. In your department, there are more than 20 employees, but only one or two promotion opportunities each year. People commonly believe that “opportunities are scarce, so everyone must compete as hard as possible.” Within the team, colleagues constantly compare their workloads and overtime hours. If someone leaves the office before 7 p.m., others tend to gossip that this person is “not motivated” or “slacking off.” Many work tasks are in fact repetitive and inefficient—designed more to “appear diligent” to supervisors than to contribute substantially to project progress. The leader also reinforces this culture, frequently posting messages such as: “The industry is highly competitive; if you don’t push yourself, you’ll be left behind. Everyone should have the mindset of ‘voluntary overtime.’” Although the overtime is said to be “voluntary,” employees who leave work on time are soon implicitly rated as “not dedicated enough” in performance evaluations. Despite investing a great deal of time and energy, you and your colleagues generally feel that your efforts yield little visible reward or personal growth, and you are unsure what real purpose this intense competition serves.

### Appendix A.2. Low Involution Condition

Please imagine that you have just joined a new company with generally attractive benefits and working conditions. In your department, there are more than 20 employees, and the company provides ample promotion and development opportunities each year. People commonly believe that “there are sufficient opportunities, so everyone can focus on doing their best.” Within the team, colleagues appreciate one another’s contributions and share resources to help each other complete projects. If someone leaves the office before 7 p.m., others tend to praise this person for “working efficiently” or “maintaining good balance.” Most work tasks are designed to be meaningful and productive—aimed at improving project quality and achieving real progress. The leader also reinforces this culture, frequently posting messages such as: “The industry rewards efficiency and innovation; working smart is more important than simply working long hours.” Although overtime is occasionally needed, it is discussed openly and compensated fairly, and leaving work on time is regarded as completely normal. As a result, you and your colleagues generally feel that your efforts bring visible achievements and personal growth, and you clearly understand the real value and purpose of your work.

### Appendix A.3. High-Level Construal Manipulation

Please recall the situation of the company you just read about. Imagine that you are working in such a department. From a long-term and abstract perspective, think about the following:

Why would you choose to work in this department?

What is the fundamental purpose and value of engaging in this kind of work in such an environment?

What might this work experience mean for your overall life direction, career goals, or personal values?

Please describe in 5–8 sentences your reflections on the reasons, meanings, and significance of doing this work from a broader and more long-term perspective.

### Appendix A.4. Low-Level Construal Manipulation

Please recall the situation of the company you just read about. Imagine that you are working in such a department. From a specific and concrete perspective, think about the following:

How would you organize your daily work schedule?

What detailed steps would you take to complete the tasks assigned by your supervisor?

In actual work, how would you interact with colleagues and supervisors, including communication, reporting, and overtime arrangements?

Please describe in 5–8 sentences your specific actions and procedures in this situation, including concrete details such as time points, tools, order of activities, frequency, and collaboration partners.
